# Supplementary material for: From sequence to enzyme mechanism using multi-label machine learning
Source: BMC Bioinformatics. 2014 May 19;15:150. doi: 10.1186/1471-2105-15-150 (PMC4229970; doi:10.1186/1471-2105-15-150)
Supplement: Additional file 2 — Java code of ml2db. Additional file ml2db_code.tar.gz contains the Java source code to run the multi-label machine learning experiments and save the results to database. The code’s Javadoc is included. [file 1471-2105-15-150-S2.zip › additional file 2/ml2db/utils/doc/uk/ac/standrews/utils/main/maputils/TableMap.html]

TableMap


JavaScript is disabled on your browser.


- Overview
- Package
- Class
- Use
- Tree
- Deprecated
- Index
- Help

- Prev Class
- Next Class

- Frames
- No Frames

- All Classes

- Summary:
- Nested |
- Field |
- Constr |
- Method

- Detail:
- Field |
- Constr |
- Method


uk.ac.standrews.utils.main.maputils

## Class TableMap

- java.lang.Object
- - uk.ac.standrews.utils.main.maputils.TableMap

- ---

    

  ```
  public class TableMap
  extends java.lang.Object
  ```

  A table map is a map containing relations [key > list of values] [value >
  list of keys]

  Version:
  :   3 Jun 2008

  Author:
  :   Luna De Ferrari luna.deferrari-at-ed.ac.uk

- - ### Constructor Summary

    Constructors

    Constructor and Description

    `TableMap()`

    Initialises the object with an empty 'one to many' map.

    `TableMap(java.util.Vector<java.lang.String> keys,
    java.util.Vector<java.lang.String> values)`

    Initialise with a list of key-value pairs
  - ### Method Summary

    Methods

    | Modifier and Type | Method and Description |
    | --- | --- |
    | `void` | `addKeyValue(java.lang.String key, java.lang.String value)` Add a key/value set to the one to many map |
    | `boolean` | `containsKey(java.lang.String key)` |
    | `boolean` | `containsKeyValue(java.lang.String key, java.lang.String value)` |
    | `boolean` | `containsValue(java.lang.String value)` |
    | `void` | `deleteKeyValue(java.lang.String key, java.lang.String value)` Delete a key-value pair from the one to many map |
    | `java.util.Set<java.lang.String>` | `getKeys()` |
    | `int` | `getKeyValuePairSize()` Get the number of key-value pairs in the map |
    | `java.util.Set<java.lang.String>` | `getKeyValues(java.lang.String key)` |
    | `OneToManyMap<java.lang.String,java.lang.String>` | `getMap()` Get the keys to values map |
    | `OneToManyMap<java.lang.String,java.lang.String>` | `getReverseMap()` Get the values to keys map |
    | `java.util.Set<java.lang.String>` | `getValues()` |

    - ### Methods inherited from class java.lang.Object

      `equals, getClass, hashCode, notify, notifyAll, toString, wait, wait, wait`

- - ### Constructor Detail


    - #### TableMap

      ```
      public TableMap()
      ```

      Initialises the object with an empty 'one to many' map.


    - #### TableMap

      ```
      public TableMap(java.util.Vector<java.lang.String> keys,
              java.util.Vector<java.lang.String> values)
      ```

      Initialise with a list of key-value pairs

      Parameters:
      :   `keys` - the list of keys
      :   `values` - the list of values (the value for each key in the keys list)
  - ### Method Detail


    - #### addKeyValue

      ```
      public void addKeyValue(java.lang.String key,
                     java.lang.String value)
      ```

      Add a key/value set to the one to many map

      Parameters:
      :   `key` -
      :   `value` -


    - #### containsKey

      ```
      public boolean containsKey(java.lang.String key)
      ```


    - #### containsKeyValue

      ```
      public boolean containsKeyValue(java.lang.String key,
                             java.lang.String value)
      ```


    - #### containsValue

      ```
      public boolean containsValue(java.lang.String value)
      ```


    - #### deleteKeyValue

      ```
      public void deleteKeyValue(java.lang.String key,
                        java.lang.String value)
      ```

      Delete a key-value pair from the one to many map

      Parameters:
      :   `key` -
      :   `value` -


    - #### getKeys

      ```
      public java.util.Set<java.lang.String> getKeys()
      ```

      Returns:
      :   the set of keys


    - #### getKeyValuePairSize

      ```
      public int getKeyValuePairSize()
      ```

      Get the number of key-value pairs in the map

      Returns:
      :   the number of key-value pairs in the map (each key can have more
          than one value)


    - #### getKeyValues

      ```
      public java.util.Set<java.lang.String> getKeyValues(java.lang.String key)
      ```


    - #### getMap

      ```
      public OneToManyMap<java.lang.String,java.lang.String> getMap()
      ```

      Get the keys to values map

      Returns:
      :   the map containing the data (each key and its values vector)


    - #### getReverseMap

      ```
      public OneToManyMap<java.lang.String,java.lang.String> getReverseMap()
      ```

      Get the values to keys map

      Returns:
      :   the map containing the data (each value and the vector of keys
          that have that value)


    - #### getValues

      ```
      public java.util.Set<java.lang.String> getValues()
      ```

      Returns:
      :   the set of values


- Overview
- Package
- Class
- Use
- Tree
- Deprecated
- Index
- Help

- Prev Class
- Next Class

- Frames
- No Frames

- All Classes

- Summary:
- Nested |
- Field |
- Constr |
- Method

- Detail:
- Field |
- Constr |
- Method
